# Facial Presentation of Primary Cutaneous Diffuse Large B-Cell Lymphoma, Leg Type: A Rare Case

**DOI:** 10.7759/cureus.92001

**Published:** 2025-09-10

**Authors:** Rami Madani, Mohamed Said, Talal Al-Assil, Steven Stone, Mohammad Omaira

**Affiliations:** 1 Internal Medicine, Western Michigan University Homer Stryker M.D. School of Medicine, Kalamazoo, USA; 2 Hematology and Medical Oncology, Bronson Methodist Hospital, Kalamazoo, USA; 3 Hematology and Oncology, Western Michigan University Homer Stryker M.D. School of Medicine, Kalamazoo, USA

**Keywords:** cutaneous b-cell lymphoma, diffuse large b-cell lymphoma, primary cutaneous diffuse large b-cell lymphoma - leg type, primary cutaneous lymphoma, r-chop chemotherapy, ulcerated facial lesion

## Abstract

An elderly woman presented with a solitary ulcerated facial lesion, which raised concern for various diagnoses, including squamous cell carcinoma, melanoma, basal cell carcinoma, or an infection. Given the lesion's unusual location, a skin biopsy was performed, and immunohistochemical analysis confirmed the diagnosis of primary cutaneous diffuse large B-cell lymphoma, leg type (PCDLBCL-LT). PCDLBCL-LT is a rare, aggressive form of lymphoma that typically manifests on the legs but can, as in this case, present in atypical sites such as the face. She was treated with a combination of rituximab, cyclophosphamide, doxorubicin, vincristine, and prednisone (R-CHOP) chemotherapy and radiation therapy, leading to a complete remission. Remarkably, after nearly 10 years, there has been no recurrence of the disease. This case highlights the importance of considering cutaneous lymphoma in the differential diagnosis for isolated, ulcerated skin lesions, even when they appear in uncommon locations, and emphasizes the potential for successful outcomes with early and appropriate treatment.

## Introduction

Diffuse large B-cell lymphoma (DLBCL), accounting for approximately 25-30% of non-Hodgkin lymphomas, typically presents as rapidly enlarging masses in nodal or extranodal sites and is generally responsive to six cycles of rituximab, cyclophosphamide, doxorubicin, vincristine, and prednisone (R-CHOP) chemotherapy [1]. When limited to the skin without evidence of systemic involvement, it is classified as primary cutaneous DLBCL, leg type (PCDLBCL-LT) [2]. 

PCDLBCL-LT is a rare but aggressive form of primary cutaneous B-cell lymphoma (CBCL), representing about 20% of CBCLs and 5% of all primary cutaneous lymphomas (PCLs) [3]. Within the CBCL spectrum, it carries the poorest prognosis, reflecting its inherently aggressive biology [3]. Although this variant most commonly affects the lower extremities of elderly patients with a median age of 75, up to 10-15% of cases can present in other cutaneous sites [4]. PCDLBCL-LT shows a female predominance and has been associated with chronic lymphedema and pre-existing vascular disease in the lower limbs [3-5]. The first-line treatment for PCDLBCL-LT is R-CHOP immunochemotherapy, analogous to systemic DLBCL management [2]. 

Clinically, it manifests as red to violaceous, often ulcerated nodules or tumors, with systemic symptoms being rare at onset [6]. At presentation, extracutaneous involvement is typically absent; however, the risk of systemic dissemination increases significantly over time, underscoring the need for early recognition and appropriate management. 

## Case presentation

**Figure 1:**
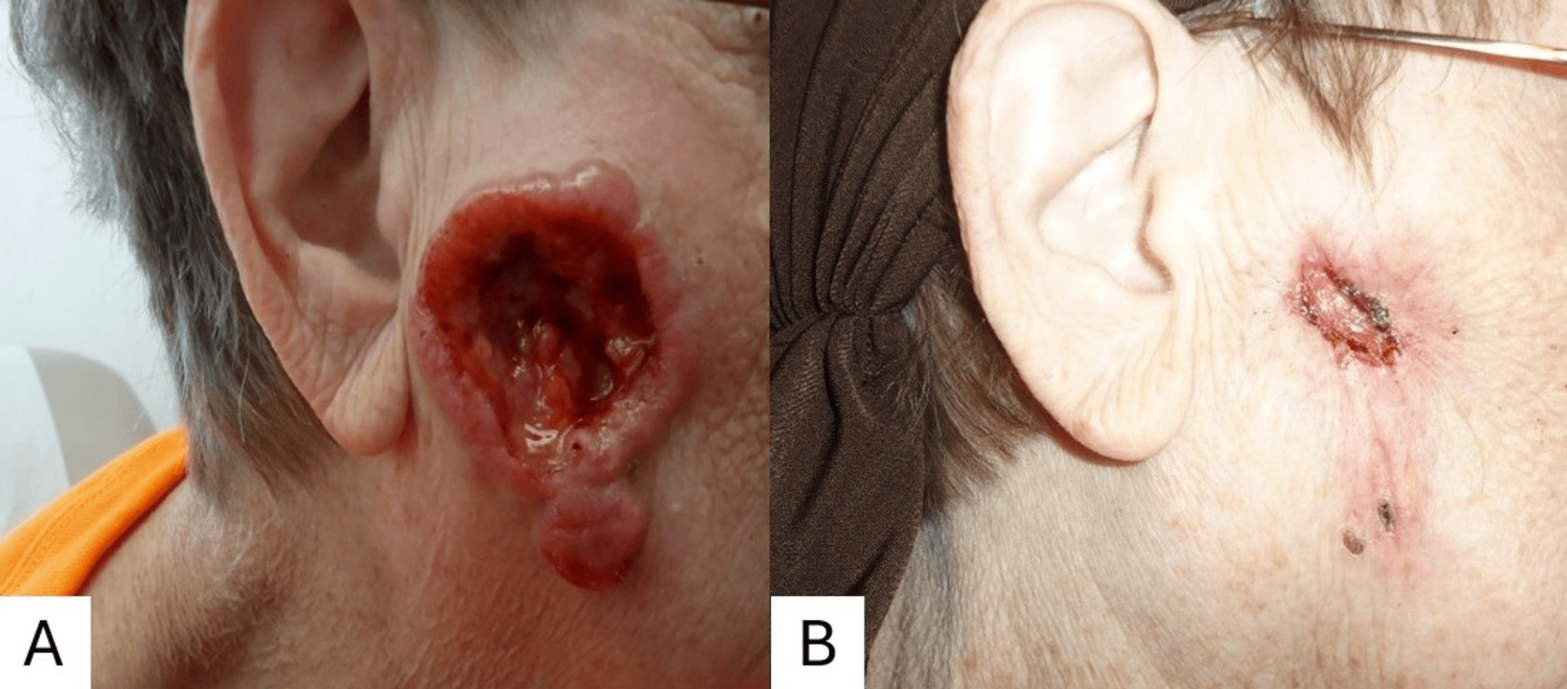
DLBCL ulcerating the superior and inferior portion of the right cheek on presentation (A) and three-month follow-up (B) after treatment with rituximab, cyclophosphamide, doxorubicin, vincristine, and prednisone followed by 30 Gy of involved-field radiation therapy. DLBCL: Diffuse large B-cell lymphoma

A woman in her 70s presented with a large, ulcerating soft tissue lesion on both the superior and inferior regions of her right cheek, with no signs of spread or similar lesions elsewhere on the body (Figure 1A). 

Initial differential diagnosis included squamous cell carcinoma, basal cell carcinoma, melanoma, and cutaneous infections. Nonmalignant considerations included sarcoidosis, cutaneous tuberculosis, syphilis, leishmaniasis, and fungal infections such as mucormycosis. However, in the absence of high-risk exposures or family history, and given the patient’s age and the sudden onset of symptoms, a malignant etiology was considered more likely. Skin biopsy was performed, and histopathological analysis revealed a dense infiltrate of poorly differentiated basaloid cells within the dermis. Immunohistochemical staining yielded positive results for leukocyte common antigen, CD20, and CD3. Markers for squamous cell carcinoma, melanoma, and endocrine tumors were all negative, including S-100, melan-A, pankeratin, high-molecular-weight keratin, CD68, CD10, CD30, synaptophysin, and chromogranin. The patient’s whole-body positron emission tomography-computed tomography (PET-CT) revealed no abnormal fluorodeoxyglucose (FDG) uptake elsewhere in her body. Her malignancy was localized to a single (I) and unique (U) site on the right cheek, and she exhibited no B-symptoms (A), such as unexplained fever, drenching night sweats, or significant weight loss. Based on these findings, the disease was staged as IAU DLBCL. 

She was treated with three cycles of R-CHOP, along with granulocyte colony-stimulating factor (G-CSF) support, followed by 30 Gy of radiation therapy in 15 fractions. Clinically, the patient demonstrated significant improvement after three cycles of chemotherapy, with near-complete resolution of the facial lesion and no evidence of local recurrence at a nine-week follow-up examination (Figure 1B). Nearly 10 years after the initial presentation, the patient shows no signs of disease recurrence. 

## Discussion

The diagnosis of PCDLBCL-LT in our patient was supported by the isolated involvement of the skin on the cheek and the absence of systemic disease, aligning with the behavior of this rare cutaneous presentation. To our knowledge, involvement of the face or cheek by PCDLBCL-LT has not been reported in the literature. 

Despite initial confinement to the skin, the subtype carries a poor prognosis, with frequent cutaneous relapse and a five-year survival rate of 40-50% [7]. Additionally, general risk factors for DLBCL may contribute to the pathogenesis of PCDLBCL-LT, including a history of B-cell-activating autoimmune diseases, hepatitis C virus infection, family history of non-Hodgkin lymphoma, and environmental or occupational exposures [8,9]. Notably, smoking has been specifically associated with an increased risk of cutaneous DLBCLs. 

Treatment consists of the R-CHOP immunotherapy regimen, generally administered every 21 days for six cycles, achieving cure rates exceeding 60%, and remains the consensus standard of care for both limited and advanced-stage disease, irrespective of molecular subtype or immunohistochemical characteristics [2]. For solitary lesions, local excision or radiotherapy may be considered; however, most patients require systemic therapy due to the aggressive nature and high risk of relapse [10]. Relapsed or refractory cases may be treated with targeted pharmacotherapy, including Bruton’s tyrosine kinase inhibitors, BCL2 inhibitors, immunomodulatory drugs, or immune checkpoint inhibitors, though these are not considered standard first-line options [8].

## Conclusions

This case highlights an uncommon cutaneous presentation of PCDLBCL-LT involving the cheek. The patient's favorable response to combined R-CHOP therapy and localized radiation, with long-term remission and no recurrence nearly 10 years later, underscores the importance of prompt diagnosis and comprehensive treatment. For clinicians, this case serves as a reminder to maintain a broad differential diagnosis when evaluating persistent or ulcerated facial lesions in elderly patients. Early biopsy and collaboration with dermatopathology are essential for establishing a timely diagnosis, particularly in cases of rare malignancies such as cutaneous lymphomas. Understanding the role of immunohistochemical markers and systemic therapy such as R-CHOP, as well as the nuances of coordinating multidisciplinary care, is vital to ensuring optimal outcomes in these aggressive but potentially curable cases. 
